# Supplementary material for: Characteristics of inclusions in chang 7 member and shale oil accumulation stages in Zhijing-Ansai area, Ordos Basin
Source: PLoS One. 2024 May 2;19(5):e0302468. doi: 10.1371/journal.pone.0302468 (PMC11065247; doi:10.1371/journal.pone.0302468)
Supplement: Attachment — Submitted filename: Response to Reviewers.docx [file pone.0302468.s001.docx]

Responses to Academic Editor

Thank you for your comments, we have studied the valuable comments from you and

tried our best to revise the manuscript.

1. Response: Completed the modification of Figure 1 to make it follow the CC BY 4.0 license

**For the suggestions and questions you put forward in the email, we have completed the correction. And will reply in the manuscript and reply letter.**

Responses to Reviewer 1

Thank you for your comments, we have studied the valuable comments from you and

tried our best to revise the manuscript. All the modified parts are marked up using

“track changes” fuction of Word. The details are as follows:

**1.Comment:**What criteria were used to select inclusions for analysis (e.g. inclusion size, shape)?

Response:Thank you for your question. According to the People 's Republic of China oil and gas industry standard SY /T 6010-1994 sedimentary rock inclusion homogenization temperature and salinity determination method, the inclusion test was carried out. In theory, any mineral crystallized from the fluid will contain inclusions. The main mineral ( quartz ) grains with good transparency and good crystallinity were selected for microscopic observation from low to high magnification.

**2.Comment:**How was the microscope stage calibrated and what was the estimated temperature uncertainty?

Response:Thank you for your question. Firstly, the microscope is placed under the light source, and the brightness and uniformity of the light source are adjusted to make the observation area have enough light and appropriate contrast. The second step is to adjust the eyepiece. Next, adjust the focal length of the eyepiece, so that the field of view of the eyepiece is clear and without distortion. It can be achieved by rotating the focusing wheel of the eyepiece. The third step is to adjust the objective lens : select the appropriate objective lens, adjust the focal length of the objective lens, so that the image of the observed sample is clearly visible. Similarly, it can be achieved by rotating the focusing wheel of the objective lens. Step 4 Calibration scale : In the field of view of the microscope, there is usually a scale to measure the length or diameter of the sample. First, a standard sample with a known length is measured under a microscope, and then the scale of the microscope is adjusted to match the standard length. The fifth step of calibrating the eyepiece micrometer is to use a standard sample with a known length to measure under the microscope, and then adjust the scale of the eyepiece micrometer to make it consistent with the standard length. Finally, check the microscope 's focusing system to ensure that clear images can be obtained under different observation conditions. The measurement error of the micro cold and hot stage instrument is ± 0.1 °C.

**3.Comment:**Have other inclusion studies in the area reported consistent or differing accumulation timings? If differing, how do the authors reconcile the interpretations?

Response:Thank you for your question. Other scholars in the Yanchang Formation of the Ordos Basin have studied the accumulation period of inclusions. The reservoir accumulation period of Mesozoic Yanchang Formation in southeastern Ordos Basin is mainly Early Cretaceous ( 93-120Ma ). This paper is aimed at the accumulation period of the Yanchang Formation in Zhijing Ansai area. Although it is affected by the region, the recovery of the accumulation period echoes with previous scholars.

Responses to Reviewer 2

Thank you for your comments, we have studied the valuable comments from you and

tried our best to revise the manuscript. All the modified parts are marked up using

“track changes” fuction of Word. The details are as follows:

1. **Comment:**The referenced literature needs to be closely integrated with the topic and paper materials, and it is recommended to use newer references.

Response:Thank you for your suggestions. I have made modifications to the questions raised and highlighted them.

2. **Comment:**It is recommended to supplement the experimental data in Table 1.

Response:Thanks for your valuable advice. All relevant data are within the paper.

3. **Comment:**It is recommended that a scale bar be added to Figure 8.

Response:Thank you for your question. I will make modifications to Figure 8 and highlight it in the manuscript.

4. **Comment:**The title "chang 7" in Figure 6 is not standard and should be changed to "chang 7 member".

Response:Thanks for pointing out the error.I have made corrections to this issue.

5. **Comment:**The marked range in Figure 4 is not clear, replace it with an obvious outline.

Response:Thanks for your valuable advice.I have made corrections to the images, please refer to the manuscript.

6. **Comment:**Finally, pay attention to the writing format of experimental data in the article.

Response:Thank you for pointing out the error. I have made modifications to the format.
